# Supplementary material for: Single-Cell RNA-Seq Identifies Pathways and Genes Contributing to the Hyperandrogenemia Associated with Polycystic Ovary Syndrome
Source: Int J Mol Sci. 2023 Jun 25;24(13):10611. doi: 10.3390/ijms241310611 (PMC10341507; doi:10.3390/ijms241310611)
Supplement: Supplementary file 1 [file ijms-24-10611-s001.zip › supplemental.figures.docx]

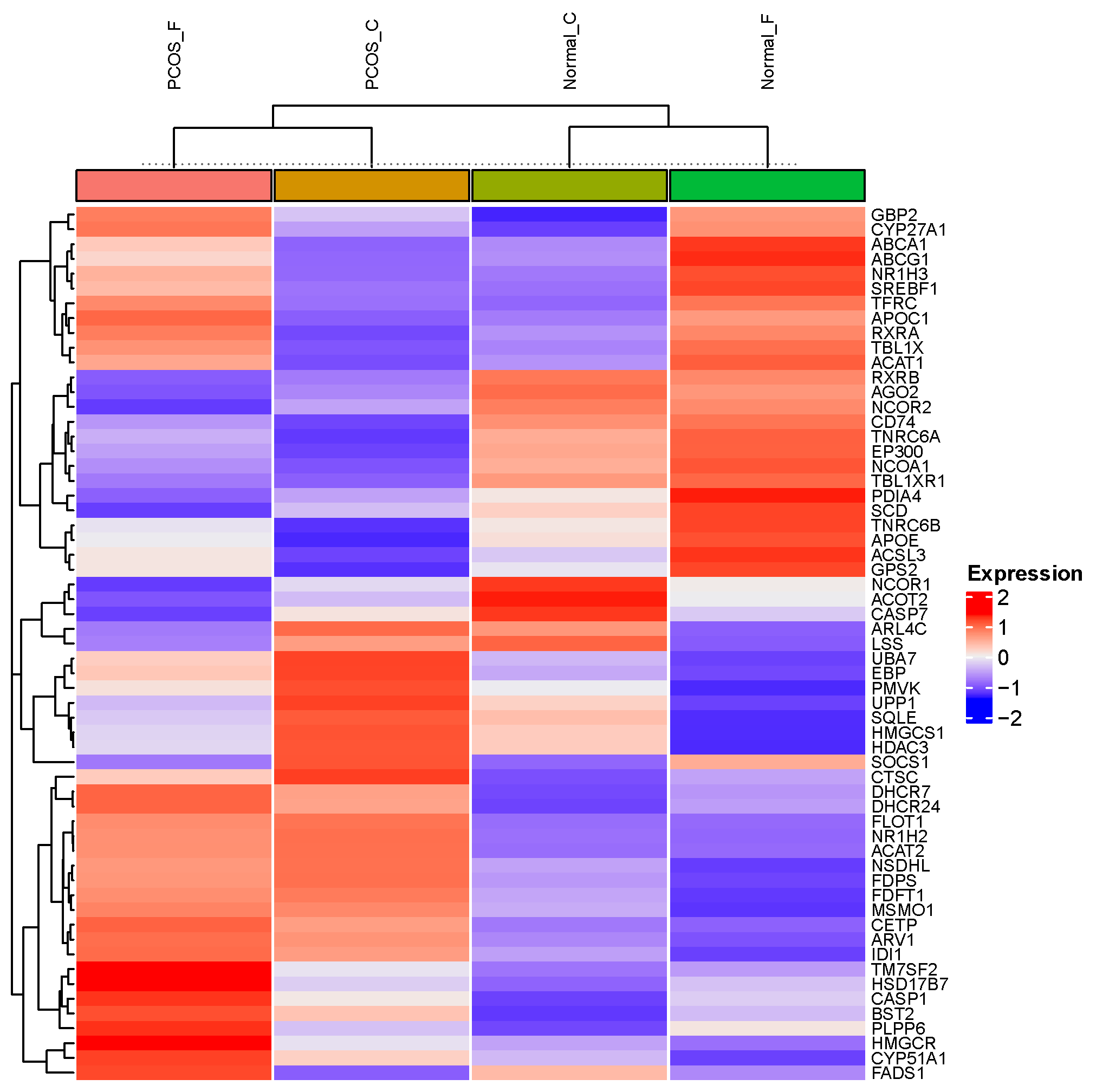


**Figure S1**. Heatmap of significant differentially expressed genes in significant differentially expressed cholesterol acquisition pathways, between normal (control, C) and PCOS theca cells treated with and without 20 mM forskolin (F) for 24 h.


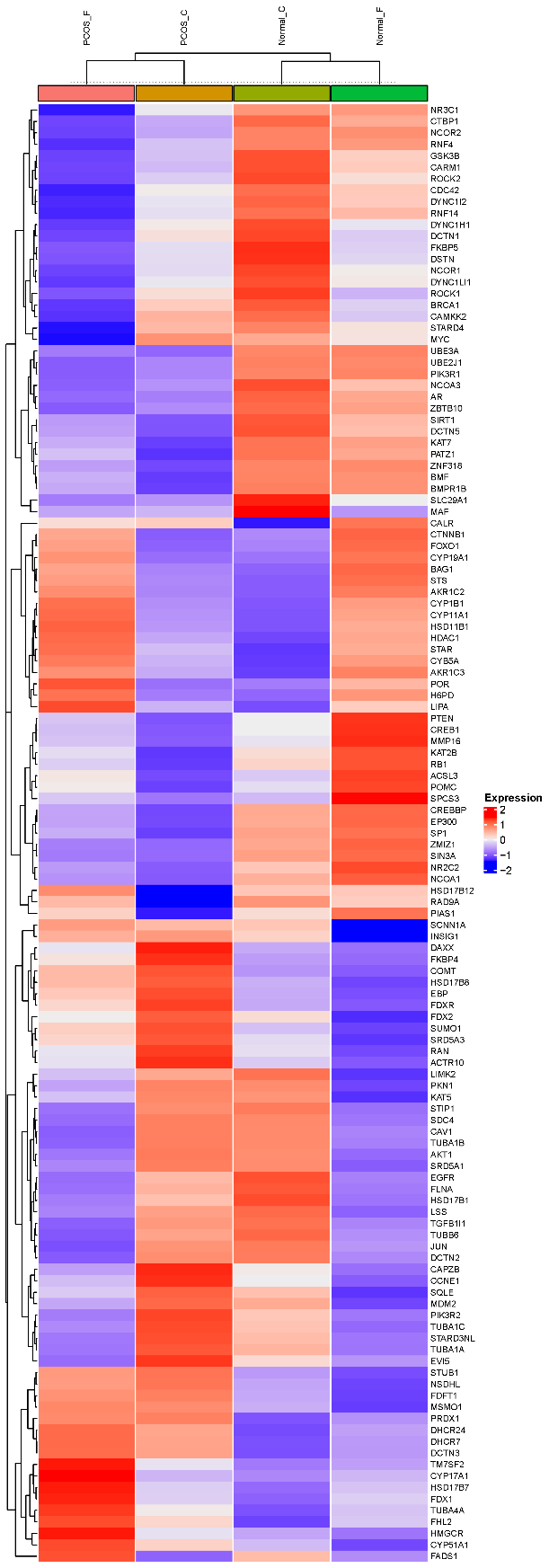


**Figure S2**. Heatmap of significant differentially expressed genes in significant differentially expressed steroidogenesis pathways, between normal (control) and PCOS theca cells treated with (F) and without (C), 20 mM forskolin (F) for 24 h.

.
